# ILSI Europe perspective review: site-specific microbiota changes during pregnancy associated with biological consequences and clinical outcomes: opportunities for probiotic interventions

**DOI:** 10.1080/19490976.2025.2501186

**Published:** 2025-05-21

**Authors:** Eduard Flores Ventura, Jonathan A. Lane, Sondra Turjeman, Nikoletta Vidra, Gisela Adrienne Weiss, Gabriele Gross, Ching-Yu Chang, Omry Koren

**Affiliations:** a Department of Biotechnology, Institute of Agrochemistry and Food Technology – Spanish National Research Council (IATA-CSIC), Valencia, Spain; b Health and Happiness (H & H) Group, H & H Research, National Food Innovation Hub, Teagasc Moorepark, Fermoy, Co., Cork, Ireland; c Azrieli Faculty of Medicine, Bar-Ilan University, Safed, Israel; d Science Department, Yakult Europe, Almere, The Netherlands; e Department Innovation, Yili Innovation Center Europe, Wageningen, The Netherlands; f Mead Johnson Nutrition Institute/Reckitt, R & D, Nutrition Science Platforms, Nijmegen, The Netherlands; g International Life Science Institute, International Life Science Institute, European Branch, Brussels, Belgium; h Kyung Hee University, Seoul, The Republic of Korea

**Keywords:** Maternal health, probiotics, pregnancy, gut microbiota, oral microbiota, vaginal microbiota, supplementation, microbiome

## Abstract

Pregnancy induces notable alterations in the gut, vaginal, and oral microbiota driven by hormonal, immune, metabolic, dietary, and environmental factors. During pregnancy, the gut microbiota is characterized by increased proportions of the genus *Bifidobacterium* and the phyla Pseudomonadota (formerly Proteobacteria) and Actinomycetota (formerly Actinobacteria). These changes occur alongside reduced alpha diversity and greater beta diversity, changes that influence maternal metabolism and fetal development. Shifts in gut and oral microbiota have been associated with complications such as preterm birth (PTB), pre-eclampsia, and gestational diabetes (GDM), though patterns are sometimes inconsistent. The vaginal microbiota remains *Lactobacillus*-dominant during pregnancy, with reduced diversity leading to reduced risk of pathogenic infection and increased diversity has been linked with a higher risk of PTB. Hormonal changes also affect the oral microbiota, potentially increasing pathogenic species and contributing to adverse outcomes like PTB. Probiotic supplementation during pregnancy has significant potential to reduce adverse pregnancy outcomes; however, clinical studies are still limited. Probiotics may be effective in alleviating maternal constipation and lead to lower PTB risk, particularly by modulating the vaginal microbiota, but they have limited impact on GDM. In the context of maternal mental health, some studies suggest benefits of probiotics in reducing anxiety, but effects on depression are inconclusive. This perspective examines how pregnancy-related microbial shifts, both natural and probiotic-induced, affect maternal and fetal health and highlights potential opportunities for the innovative use of probiotics during the gestation period.

## 1. Introduction

The maternal microbiota, defined by the International Scientific Association for Probiotics and Prebiotics (ISAPP) as “the microorganisms themselves: bacteria, archaea, lower and higher eukaryotes, and viruses in a defined environment”, in this case, within the mother, undergoes significant changes during pregnancy. These changes can influence both maternal and fetal health.^1–3^ Alterations in maternal gut, vaginal, and oral microbiota during the first, second, and third trimesters have been extensively studied elsewhere, and biological consequences and clinical outcomes, such as gestational diabetes mellitus (GDM), pre-term birth (PTB), and pre-eclampsia (PE) have been associated with such changes.^4–6^ Overall, the specific biological mechanisms and microbial taxa involved remain under investigation.

Microbiome-modulating interventions during pregnancy aim to support healthy biological changes, such as hormonal, immune, and metabolic adjustments that occur during and throughout pregnancy and may significantly prevent adverse pregnancy outcomes. Such interventions can include the use of “biotics”, such as prebiotics, probiotics, and postbiotics, or dietary change. The use of commercially available probiotic(s) during pregnancy is one such interventions that has been clinically studied, however with inconsistent results. Although some studies suggest that probiotics, defined as “live microorganisms that, when administered in adequate amounts, confer a health benefit on the host”,^6^ might improve pregnancy outcomes, these associations are based on limited and primarily inferential evidence. The reasons for variability in outcomes across studies can be associated with clinical study design, use of different probiotic strain(s) and doses, and/or the lack of precision interventions such as not factoring when/how a probiotic should be consumed or how host genetics (e.g. secretor status) may influence colonization potential.^7,8^ The efficacy and safety of probiotics can vary significantly due to strain-specific effects. Different strains of the same species can cause varied clinical outcomes, underscoring the complexity of recommending probiotics for specific uses.^9^ Genetic variation between strains influences key characteristics such as their metabolic activities, their ability to adhere to epithelial cells, and their immunomodulatory properties.^10^ These factors can directly impact the effectiveness and safety profile of probiotic interventions. Moreover, the effective dosage of probiotics and time of prescription are highly heterogeneous, influenced by variables such as the viability of the strain, the formulation used, and the individual’s existing commensal microbial composition.^11^ This variability suggests that a personalized approach might be necessary to optimize probiotic interventions, further complicating standardization and clinical application.

Taken together, these challenges highlight the need for a review that not only maps the knowledge on microbial shifts during pregnancy and their relationship with maternal and fetal health but also examines probiotic efficacy and safety in this context. We aimed to identify gaps and opportunities, along with exploring the potential for personalized probiotic interventions. By understanding these dynamics, we can better design and utilize probiotics to enhance gestational health. Rather than systematically reviewing all existing data, this perspective aims to provide an overview of emerging trends and potential probiotic interventions during pregnancy, offering a targeted and innovative synthesis. We then give a perspective on a more targeted approach to the use of probiotics during pregnancy that can support future clinical study design and the subsequent use of probiotics to prevent adverse pregnancy outcomes.

## 2. Maternal microbiota changes and biological consequences associated with clinical outcomes during pregnancy

**Figure 1.**
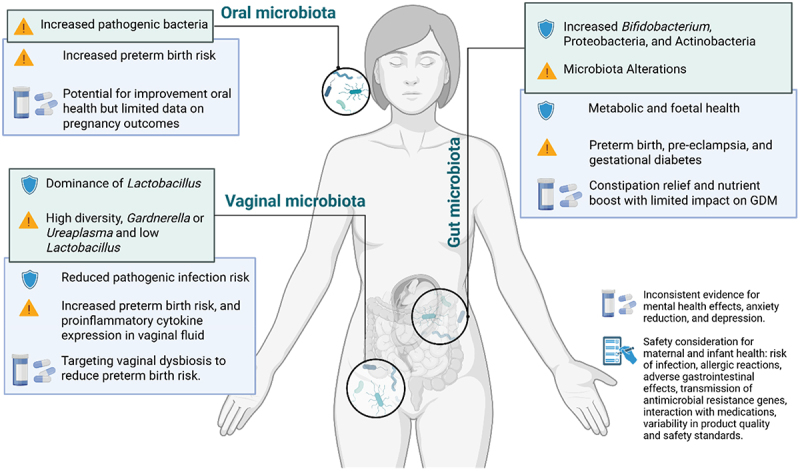
Maternal microbiota shifts during pregnancy and their clinical implications. Overview of microbiota changes in the oral, vaginal, and gut compartments during pregnancy, and their potential impact on maternal and foetal outcomes, including preterm birth, infection risk, metabolic health, and gestational complications. Symbols indicate: Blue shield, microbial shifts associated with beneficial health effects; yellow triangle, microbial shifts associated with adverse health effects; green boxes, changes in endogenous maternal microbiota; blue boxes, associated biological implications; capsule symbol, potential effects of probiotic interventions. Figure created with BioRender.com.© Flores, V.E. (2025). Retrieved from https://BioRender.com/p25dwhp.

Major changes in microbial communities during pregnancy and their implications for maternal or fetal health are highlighted in Figure 1. During pregnancy, the gut microbiota undergoes significant changes between the first and third trimester, influenced by physiological factors such as increased progesterone levels.^12^ In the first trimester, a lower abundance of *Bifidobacterium* and *Streptococcus* has been associated with PTB. Meanwhile, PE and GDM have been associated with decreased abundance of short-chain fatty acid (SCFA)-producing bacteria and altered metabolic pathways. In the third trimester, pregnant women display a higher proportion of *Bifidobacterium*, as well as the phyla Pseudomonadota (formerly Proteobacteria) and Actinomycetota (formerly Actinobacteria). This shift is accompanied by reduced alpha diversity, increased beta diversity and a rise in opportunistic pathogens compared to the first trimester. These microbiota alterations impact maternal metabolism and fetal organ development.^13,14^ Changes in the gut microbiota during the third trimester have been linked via statistical inference to PTB, PE, and GDM, characterized by lower diversity and specific bacterial imbalances.^15–17^

Also, the vaginal microbiota changes significantly throughout pregnancy toward a predominance of *Lactobacillus spp*., including *Lactobacillus crispatus*, *Lactobacillus iners*, *Lactobacillus gasseri*, and *Lactobacillus jensenii*, which produce lactic acid to inhibit pathogen growth. This shift results in reduced microbial diversity, which is crucial for preventing bacterial vaginosis and sexually transmitted infections. Postpartum, microbial diversity increases, and *Lactobacillus* abundance decreases.^18,19^ Additionally, a study that analyzed weekly vaginal samples throughout gestation found that highly diverse vaginal communities and high levels of *Gardnerella* or *Ureaplasma*, combined with low levels of *Lactobacillus*, were associated with increased risk of PTB and proinflammatory cytokine expression in vaginal fluid, which may trigger PTB.^20^

The oral microbiota, dominated by *Streptococcus spp*., undergoes significant shifts during pregnancy due to hormonal changes, with increases in pathogenic bacteria like *Prevotella intermedia*, *Porphyromonas gingivalis*, and *Fusobacterium nucleatum*.^21^ These shifts, linked to changes in estrogen, progesterone, and other hormones, can lead to adverse pregnancy outcomes by promoting systemic inflammation.^22^ Periodontal disease-associated bacteria such as *F. nucleatum* and *P. gingivalis* are notably linked to PTB and PE,^23^ while oral microbiota with increased abundance of *Neisseria* and *Leptotrichia* are correlated with GDM.^24^

## 3. Clinical effects of probiotic interventions during pregnancy

### 3.1. Maternal group B streptococcal (GBS) infection

#### 3.1.1. Summary of key clinical trials

To date, there have been more than seven clinical studies investigating the impact of probiotics on potential maternal infection during pregnancy, involving over 700 women (Table 1). In these studies, three different probiotic supplements have been used (I) *Lacticaseibacillus rhamnosus* (GR-1®) and *Limosilactobacillus reuteri* (RC-14®), (II) *L. jensenii* (LBV 116™), *L. crispatus* (LBV88™), *L. rhamnosus* (LBV 96™), and *L. gasseri* (LBV 150N™), and (III) *Ligilactobacillus salivarius* (CECT 9145).^25–29,53^

**Table 1.** Tested probiotics in clinical trials during pregnancy.

Probiotic strains tested in clinical trials during pregnancy	Sample size (Placebo/Probiotic)	Dose (CFU)	Trimester of adm	Duration and type of adm		Microbiota analyzed	Observed microbiota changes	Outcomes	References
**GBS Infection**									
*L. jensenii* (LBV 116™), *L. crispatus* (LBV88™), *L. rhamnosus* (LBV 96™) and *L. gasseri* (LBV 150GG®)	41/41	2 x 10^8^	3rd trimester	14 d	Oral	Vaginal	No significant vaginal microbiota shifts observed	Probiotic treatment (63.6% vs. 77.8% persisting GBS positivity after treatment in the verum and placebo groups, respectively.	Farr et al.,^25^
*L. rhamnosus* (GR-1®) and *L. reuteri* (RC-14®)	144/148	1 x 10^8^	1st trimester	8 wk	Oral	Vaginal (Nugent score)	No improvement in vaginal flora; slight decrease in normal flora in both groups	The proportion of normal vaginal microbiota decreased from 82.6% to 77.8% in the treatment group and from 79.1 to 74.3% in the placebo group, with no significant difference across groups after intervention.	Gille et al.,^26^
*L. rhamnosus* (GR-1®) and *L. reuteri* (RC-14®)	50/49	1 x 10^8^	2nd trimester	3 wk	Oral	Vaginal and rectal	↓ GBS colonization: 42.9% (probiotic) vs 18% (placebo)	The GBS colonization results changed from positive to negative in 21 women in the probiotic group (42.9%) and in nine women in the placebo group (18.0%) during this period.	Ho et al.,^27^
*L. rhamnosus* (GR-1®) *and L. reuteri* (RC-14®)	13/19	1 x 10^8^	3rd trimester	3 wk	Oral	Vaginal	↑ *Lactobacillus* spp. in those completing ≥14 d; no GBS change	In the control group of 3/13 participants were GBS negative at the time of their final swab collection. In the probiotics group of 4/19 participants, were GBS negative at the time of their final swab collection. No significant decrease in GBS in their vaginas.	Olsen et al.,^28^
*L. salivarius* (CECT 9145)	14/25	9 x 10^10^	2nd trimester	12 wk	Oral	Vaginal & Rectal (with sequencing)	↓ *Streptococcus agalactiae*; ↑ *Lactobacillus* dominance (*L. salivarius*) in probiotic group	72% and 68% of the women in the probiotic group were GBS-negative in the rectal and vaginal samples, respectively.	Martín et al.,^29^
**Constipation**									
*B. bifidum* (W23), *B. animalis* subsp. *lactis* (W52), *B. longum* (W108), *L. casei* (W79), *L. plantarum* (W62) and, *L. rhamnosus* (W71)	0/20	4 x 10^9^	2nd trimester	4 wk	Oral	NA	NA	Post-intervention, defecation frequency significantly increased from 3.1 at baseline to 6.7. A significant decrease in sensation of anorectal obstruction from 90.0% to 45.0%, sensation of incomplete evacuation from 90.0% to 40.0%, straining during defecation from 100% to 65%, episodes of abdominal pain from 60% to 20%, and the presence of reflux episodes from 60% to 20%.	de Milliano et al.,^30^
Yoghurt enriched with *B. animalis* subsp. *lactis* (BB-12®) and *L. acidophilus* (LA-5®) Yoghurts contained *L. bulgaricus* and *S. thermophilus.*	29/28	4.8 x 10^10^	2nd trimester	4 wk	Oral	NA	NA	Constipation symptoms including straining, anorectal obstruction, manipulation to facilitate defecation, consistency of stool and colour of stool were improved significantly in both groups. No statical differences between the groups.	Mirghafourvand et al.,^31^
*L. rhamnosus* (GR-1®) *and L. reuteri* (RC-14®)	152/152	2.5 × 10^9^ (each strain)	1st trimester	26 wk	Oral	Vaginal (Nugent and sequencing)	No change in diversity, bacterial vaginosis rates, or colonization by probiotic strains	No significant changes, nor differences between groups.	Husain et al.,^32^
**Nutrient Status**									
Probiotic yoghurt enriched with *L. acidophilus* (LA5) and *B. lactis* (BB12)	33/37	1 × 10^7^	3rd trimester	9 wk	Oral	NA	NA	Consumption of probiotic yogurt resulted in maintaining serum calcium levels compared with the conventional yogurt. Within-group differences in the conventional yogurt group revealed a significant reduction of serum calcium levels. No significant differences were found between the two yogurts in terms of their effects on serum iron, AST and ALT levels.	Asemi and Esmaillzadeh^33^
*L. plantarum* 299v (LP299V®)	110/118	1 x 10^10^	1st trimester	25 wk	Oral	NA	NA	Attenuated decrease in serum ferritin from baseline to week 28 and week 35 and resulted in reduced prevalence of iron deficiency (59% vs 78%, and iron deficiency anemia (7.4% vs 21%, at week 35. Intake of probiotic also resulted in beneficial effects on the soluble transferrin receptor and total body iron at week 35.	Axling et al.,^34^
*L. acidophilus, L. casei, B. bifidum*, and *L. fermentum*	24/24	2 × 10^9^	2nd trimester	6 wk	Oral	NA	NA	Probiotic supplementation significantly decreased fasting plasma glucose, serum insulin levels, and insulin resistance, and significantly increased insulin sensitivity, compared with the placebo. Supplementation also decreased triglycerides, VLDL-cholesterol, and total-/HDL-cholesterol ratio and significantly increased HDL-cholesterol levels compared with the placebo. Furthermore, led to a significant reduction in plasma malondialdehyde, and a significant elevation in plasma nitric oxide and total antioxidant capacity was observed compared with the placebo.	Babadi et al.,^35^
*L. plantarum* 299v (LP299V®) and 5 mg of iron as ferrous lactate.	0/21	1 x 10^9^	Not pregnant(reproductive-age women)	2 wk	Oral	NA	NA	Mean iron absorption from the drink was significantly higher than the iron absorption from the control drink.	Hoppe et al.,^36^
*L. plantarum* 299v (LP299N™)	0/42	1 x 10^10^	Not pregnant (reproductive-age women)	4 wk	Oral	NA	NA	The absorption of iron was significantly higher in the probiotic group.	Hoppe et al.,^37^
Vitamin D and *L. acidophilus, B. bifidum, L. reuteri*, and *L. fermentum*	28/29	8 × 10^9^	Late 2nd–early 3rd trimester	6 wk	Oral	NA	NA	Vitamin D and Probiotic group showed significantly reduced, fasting plasma glucose, serum insulin levels, and homeostasis model of assessment-insulin resistance, and significantly increased the quantitative insulin sensitivity check index. Probiotic group also showed significantly reduced triglycerides, HDL-/total cholesterol ratio, high sensitivity C-reactive protein, and malondialdehyde, also, an increase of HDL-cholesterol, and total antioxidant, when compared to placebo.	Jamilian et al.,^38^
*L. rhamnosus* (V®) and *B. animalis* subsp *lactis* (BB-12®)	20/10	1 x 10^9^	From 1st trimester (~13.8 weeks)	NA	Oral	NA	NA	The major differences in placental fatty acids were attributable to a higher concentration of omega-3 fatty acids in both intervention arms than in controls. Probiotic and diet group had higher concentrations of linoleic and dihomo-γ-linolenic acids compared with dietary counselling with placebo or controls.	Kaplas et al.,^39^
*L. plantarum* 299v (LP299V®)	6/7	1 x 10^10^	2nd trimester	17.5 wk	Oral	NA	NA	A slower decline in maternal haematological and iron parameters across pregnancy was observed in the probiotic group compared to placebo.	OjiNjideka Hemphill et al.,^40^
**GDM**									
*L. salivarius* (UCC118)	75/74	1 x 10^9^	Mid-late pregnancy	8 wk	Oral	NA	NA	No differences between the probiotic and placebo groups in postintervention fasting glucose. Among 100 women managed with diet and exercise alone, fasting plasma glucose decreased significantly within both the probiotic and placebo groups, but the levels between groups did not differ.	Lindsay et al.,^41^
*L. rhamnosus* (V®) and *B. animalis* subsp *lactis* (BB-12®)	85/85	1 x 10^10^	1st trimester onward	17 wk	Oral	NA	NA	The risk of GDM was significantly reduced in the diet/probiotics group when compared to the control group.	Luoto et al.,^42^
*L. rhamnosus* (HN001) and *B. animalis subsp. lactis* (420), taken with fish oil.	110/110	1 x 10^10^	2nd trimester	26 wk	Oral	NA	NA	No significant difference in the incidence of GDM between the intervention groups.	Pellonperä et al.,^43^
*L. rhamnosus* (GG®) and *B. animalis* subsp *lactis* (BB-12®), and 2g Myoinositol	98/48	5 x 10^8^	Early-mid pregnancy	15 wk	Oral	NA	NA	Significant decrease in GDM incidence in the probiotic group.	Reyes-Muñoz et al.,^44^
*L. acidophilus* (LA1), *B. longum* (sp54 cs), and *B. bifidum* (sp9 cs)	271/271	>7.5×10^9^ > 1.5×10^9^ > 6×10^9^	2nd trimester	14 wk	Oral	NA	NA	There was no significant difference between the intervention and the control group regarding FBG, OGTT-1h and OGTT-2h. The incidence of GDM in the intervention group was 41.9% which was not significantly different from the control group (40.2%).	Shahriari et al.,^45^
**Mental Health**									
*Bifidobacterium bifidum* (W23), *Bifidobacterium lactis* (W51), *Bifidobacterium lactis* (W52), *Lactobacillus acidophilus* (W37), *Lactobacillus brevis* (W63), *Lactobacillus casei* (W56), *Lactobacillus salivarius* (W24), *Lactococcus lactis* (W19) and *Lactococcus lactis* (W58)	20/20	5 x 10^9^	Late 2nd to 3rd	10 wk	Oral	NA	NA	Overall, there were no significant differences between groups, except for lower depressive symptoms in the probiotic group and reduced depression-related cognitive reactivity to sad mood (LEIDS-R) in the placebo group.	Browne et al.,^46^
*L. rhamnosus* (V®) and *B. animalis subsp lactis* (BB-12®)	76/88	6.5 x 10^9^	2nd trimester	20 wk	Oral	NA	NA	No significant difference between groups.	Dawe et al.,^47^
*L. rhamnosus* (HN001) and *B. animalis* subssp. *lactis* (420)	137/127	1 x 10^10^	Early pregnancy	10 mo	Oral	NA	NA	Higher depressive symptoms during pregnancy and at 12 months postpartum compared to the placebo group.	Hulkkonen et al.,^48^;
*L. rhamnosus* (HN001)	211/212	6 x 10^9^	2nd trimester to postpartum	15 wk	Oral	NA	NA	Mothers in the probiotic treatment group reported significantly lower scores for depression and anxiety postpartum compared to the placebo group.	Slykerman et al.,^49^
**Preterm Birth**									
*L. crispatus* (CTV-05)	2190/61	2 x 10^9^	2nd trimester	6 wk	Vaginal	NA	NA	The rate of early PTB <34 weeks in the probiotic group was 3.3% compared to 7% in a historical cohort of women at similar background PTB risk with no probiotic administration.	Bayar et al.,^50^;
*L. casei rhamnosus* (Lcr regenerans)	79/59	1 x 10^7^	1st trimester	8 d	Vaginal	Vaginal (Nugent score)	↓ Nugent score only in women without baseline *lactobacilli*	No significant differences in preterm birth rates between groups.	Petricevic et al.,^51^
*L. rhamnosus* (GR-1®) and *L. reuteri* (RC-14®)	32/34	2.5 × 10^9^ (each strain)	1st to 3rd trimester	12 wk	Oral	Vaginal (Nugent, sequencing)	No change in diversity or Nugent score; *Lactobacillus iners*, *G. vaginalis* dominant.	No significant differences in premature birth.	Yang et al.,^52^

CFU – Colony Forming Units; adm – Administration; NA – Not Available; RCT – Randomised Controlled Trial; GBS – Group B *Streptococcus*; Nugent score – Nugent score; LEIDS-R – Leiden Index of Depression Sensitivity – Revised; PTB – Preterm Birth; FBG – Fasting Blood Glucose; OGTT-1h/OGTT-2h – Oral Glucose Tolerance Test at 1 hour/2 hours; GDM – Gestational Diabetes Mellitus; AST – Aspartate Aminotransferase; ALT – Alanine Aminotransferase; VLDL – Very Low-Density Lipoprotein; HDL – High-Density Lipoprotein.

Studies with oral supplementation of *L. rhamnosus* (GR-1®) and *L. reuteri* (RC-14®) were randomized placebo controlled clinical trials with more than 450 women with primary and secondary outcomes associated with vaginal microbiota shifts including GBS colonization, pruritus, vaginal discharge, and pregnancy outcome including preterm delivery.^26–29,53^

Although the probiotic strains used in these studies were obtained from the same source, the probiotic dose and intervention period differed. Gille et al.^26^ reported a non-significant decrease, rather than an increase, in the proportion of normal vaginal microbiota as measured by the Nugent score, a Gram staining scoring system that assesses the balance of *Lactobacillus*, *Gardnerella*, and *Mobiluncus* to classify vaginal microbes. Similar findings were also reported by Farr et al. (2019) when studying dietary supplementation with four viable strains; 2 × 10^8^ *L. jensenii* LBV 116™ 10^11^ colony forming units (CFU)/g (DSM 22,566); 1 × 10^8^ *L. crispatus* LBV88™ 10^11^ CFU/g (DSM 22,566); 1 × 10^8^ *L. rhamnosus* (LBV 96™) 10^11^ CFU/g (DSM 22,560); 3 × 10^8^ *L*. (LBV 150N™) 10^11^ CFU/g (DSM 22,583).^26^

#### 3.1.2. Intervention duration and dose variability

**Figure 2.**
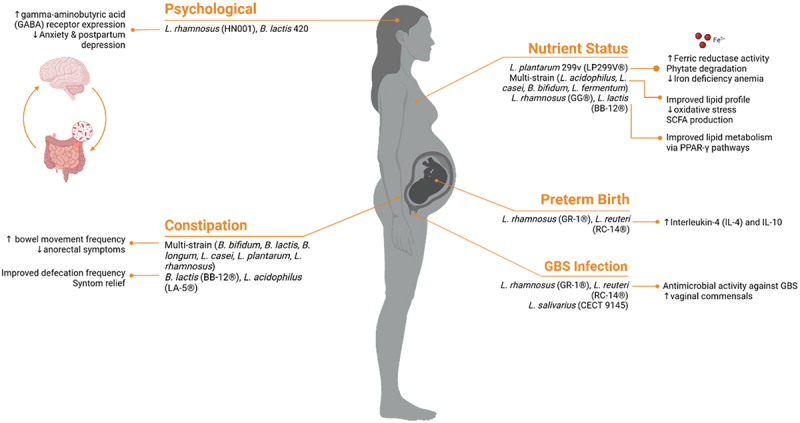
Probiotic supplementation during pregnancy and its mechanisms of impact on maternal health, including effects on mood, nutrient status, preterm birth, Group B *Streptococcus* (GBS) infection, and constipation. Figure created with BioRender. Flores, V.E. (2025) https://BioRender.com/p25dwhp.

Ho et al. (2016)^27^ demonstrated that the probiotic intervention could reduce vaginal and rectal GBS colonization rates in pregnant women and proposed that oral probiotics should be administrated early in pregnancy to reduce GBS colonization at 35–37 weeks of gestation. Olsen et al.^28^ reported that this probiotic intervention had no significant impact on vaginal GBS rates; however, they demonstrated a significant increase in vaginal commensals in the probiotic group. Additionally, a prospective study with oral supplementation of *L. salivarius* (CECT 9145) in a total of 57 pregnant women (39 rectal and vaginal *S. agalactiae*/GBS-positive women; 18 rectal and vaginal GBS-negative women) was conducted to determine the impact of the intervention on GBS eradication.^29^ Interestingly, the isolation and identification of the candidate probiotic strain for this human intervention study were based on target mechanisms of action, namely anti-microbial activities against GBS (Figure 2).

#### 3.1.3. Limitations of GBS probiotic intervention trials

Although the perspective study had a limited number of participants, it was successful in demonstrating a significant decrease in GBS colonization during pregnancy. Key limitations include the short and variable durations of probiotic exposure, which ranged from 14 d^25^ to 3 weeks,^27,28^ while others extended to 8^26,27^ and 12 weeks^29^ cross studies. Only three studies were identified as replicates in terms of probiotic strain and dosage, all of which focused on GBS as an outcome. These studies^26–28^ investigated *L. rhamnosus* (GR-1®) and *L. reuteri* (RC-14®), administered at 10^8^ CFU/g.

In conclusion, current evidence suggests a potential for *Lactobacillus* probiotic interventions to reduce the risk for maternal GBS infection, when taking into account antimicrobial activity against GBS and when applied in the most effective time window during gestation, specifically, a 3-week treatment in the second trimester.^27^

### 3.2. Maternal constipation

#### 3.2.1. Small-scale study example

A four-week pilot study assessing the administration of a multi-strain formulation containing *Bifidobacterium bifidum* (W23), *Bifidobacterium animalis* subsp. *lactis* (W52), *Bifidobacterium longum* (W108), *Lacticaseibacillus casei* (W79), *Lactiplantibacillus plantarum* (W62) and *L. rhamnosus* (W71) during pregnancy showed a significant increase in bowel movement frequency in 20 constipated women. There was also a significant decrease in the presence of sensation of incomplete evacuation, sensation of anorectal obstruction, straining during defecation, abdominal pain, and reflux episodes, but no effect on hard stools and manual maneuvers. No side effects were reported.^30^

#### 3.2.2. Yogurt-based intervention study

In addition, in a randomized controlled trial (RCT), a treatment group of pregnant women received 300 g of yogurt enriched with *B. animalis* subsp. *lactis*, (BB-12®) and *Lactobacillus acidophilus* (LA-5®) 4.8 × 10^10^ CFU, while the control group consumed conventional yoghurt without added probiotics (*n* = 30) for 4 weeks. Both probiotic and conventional yogurts contained *Lactobacillus bulgaricus* and *Streptococcus thermophilus*. In both groups, a significant increase in frequency of defecation and amount of stool was observed. Symptoms such as straining and sensation of anorectal obstruction decreased, whereas the need for manipulation to facilitate defecation increased significantly in both groups. No significant effect on the sensation of incomplete evacuation after defecation was observed.^31^

#### 3.2.3. Limitations of current studies

None of these studies measured mechanistic parameters. Furthermore, generalizability to the broader population remains limited. For example, one study on constipation included only cases (i.e. no control group),^30^ precluding between-group comparisons, and had a duration of just 4 weeks.^30^ In contrast, the study with the largest sample size, 152 participants in both the treatment and placebo groups,^32^ also had a long treatment duration (26 weeks). However, despite its scale, it failed to identify significant differences or changes between groups.

In conclusion, although the effects of probiotic interventions on maternal constipation have been described, the overall efficacy and benefits are inconsistent.

### 3.3. Maternal nutrient status

#### 3.3.1. Effects of specific probiotic strains

Several clinical trials demonstrated significant effects of various probiotic supplements on maternal nutrient status, including *L. plantarum* 299 v (LP299V®), multi-strain probiotic capsules containing *L. acidophilus*, *L. casei*, *B. bifidum*, and *Limosilactobacillus fermentum* (2 × 10^9^ CFU/g each),^54^ capsules containing *L*. *rhamnosus* (GG®) (ATCC 53,103; Valio, Helsinki, Finland) and *B. animalis* subsp. *lactis*, (BB-12®) (Chr. Hansen, Hørsholm, Denmark) 10^9^ CFU/day, and probiotic-enriched foods, with no adverse effects.^39^

#### 3.3.2. Iron absorption and anemia

LP299V® administration was shown to positively impact dietary iron bioavailability and reduce the prevalence of iron deficiency anemia early in pregnancy.^34,36,37,40^ Mechanistic insights suggest that LP299V® increases ferric iron (Fe^3+^) levels in digested meals, potentially by affecting ferric reductase activity and phytate degradation (Figure 2). Only one trial reported adverse effects, which may have been due to the combination of iron supplementation and LP299V.^34^

#### 3.3.3. Lipid metabolism and oxidative stress

In addition, two probiotic intervention studies with multi-strain probiotic supplementation, one containing *L. acidophilus*, *L. casei*, *B. bifidum*, and *Limosilactobacillus fermentum* (2 × 10^9^ CFU/g each) for 6 weeks in a 24/24 placebo/treatment group, and the other with *L. rhamnosus* (GR-1®), and *B. animalis* subsp. *lactis* (BB-12®) at 10^9^ CFU/g in a 20/10 placebo/treatment during pregnancy, significantly improved lipid profiles and reduced oxidative stress.^35,39^ The suggested underlying mechanisms of action include regulation of peroxisome proliferator activated receptor gamma (PPAR-γ) gene expression, modulation of toll-like receptor-mitogen-activated protein kinase-PPAR-γ signaling pathways, and increased production of SCFAs, which affect various metabolic pathways (Figure 2).

Further, there may be effects on the placenta: multi-strain probiotics may modify placental phospholipid fatty acids and influence systemic effects through similar signaling pathways as fatty acids, including soluble cluster of differentiation 14 (CD14) and proinflammatory cytokines.^35,39^

#### 3.3.4. Combined micronutrient and probiotic supplementation

Additionally, encapsulated micronutrients and probiotic supplementation were included in a clinical trial with pregnant women receiving Vitamin D and probiotic co-supplementation containing 8 × 10^9^ CFU/day of *L. acidophilus, B. bifidum, L. reuteri*, and *L. fermentum*, strains not indicated. Vitamin D and probiotic co-supplementation significantly reduced triglyceride levels compared to the placebo, and a similar but smaller reduction in triglycerides was observed in the probiotic-only group. This might be mediated by modulation of gene expression related to lipid metabolism, suppression of toll-like receptor signaling pathways, and increased production of SCFAs.^38^

#### 3.3.5. Calcium absorption

Furthermore, consumption of a probiotic yogurt enriched with *L. acidophilus*, (LA-5®) and *B. animalis* subsp. *lactis* (BB-12®) 1 × 10^7^ CFU/ml (a total of 200 × 10^7^ CFU/day) by pregnant women helped to maintain serum calcium levels compared to conventional yogurt, possibly by enhancing calcium solubility and increasing calcium absorption.^33^

#### 3.3.6. Study design limitations and overall findings

It is important to note that these are unique studies, so direct comparison between them is not possible. While some studies included larger sample sizes, for example, 110 and 118 participants in the placebo and intervention groups, respectively, with an extended exposure period of 25 weeks,^34^ others involved notably smaller cohorts, shorter treatment durations, and differed in dosages and probiotic strains.

Overall, these findings suggest direct improvement of nutrient absorption and indirect modulation of the physiological mechanisms contributing to increased nutrient absorption and utilization by probiotics during pregnancy.

### 3.4. Gestational diabetes mellitus

#### 3.4.1. Early clinical trials

To date, seven clinical trials examining the effects of probiotics on GDM have been conducted. One of the first, a double-blind RCT of pregnant women with impaired glucose tolerance or GDM, was published over a decade ago and revealed that daily consumption of *L*. *salivarius* (UCC118) at a target dose of 10^9^ CFU did not reduce fasting blood glucose levels nor change metabolic parameters and pregnancy outcomes. Importantly, in both arms (placebo, probiotic), pre-intervention blood glucose was significantly higher than post-intervention showing that the mandated lifestyle guidelines for diet and exercise are effective for managing GDM, and probiotic treatment does not mediate or amplify these effects.^41^ In a second historic study from 2010, intensive dietary counseling provided by a nutritionist, combined with a probiotic supplement, significantly decreased the frequency of GDM compared to a control group without dietary intervention. However, no direct comparison was made with the participant group that received dietary intervention and a placebo.^42^

#### 3.4.2. Recent RCTs with null findings

More recent studies present similar findings. Two double-blind RCTs in overweight and obese pregnant women supplemented with *L. rhamnosus* (GG®) and *B. animalis* subsp. *lactis*, (BB-12®) at a dose of >10^9^ CFU showed no effect on GDM or other maternal and neonate markers.^43,45^ A double-blind RCT with *L. rhamnosus* (HN001) and *B. animalis* subsp. *lactis* (420) (10^10^ CFU each), taken with or without fish oil, did not show any effects compared to placebo,^43^ and also a fourth study, with *L*. *acidophilus* (LA1) (>7.5 × 10^9^ CFU), *B. longum* (sp54 cs) (>1.5 × 10^9^ CFU), and *B. bifidum* (sp9 cs) (>6 × 10^9^ CFU) did not demonstrate an effect.^45^ A final study examining supplementation of *L. rhamnosus* (HN001) (6 × 10^9^ CFU) in pregnant women with personal or partner history of atopic disease found a positive effect on GDM in women >35 yrs and those with a history of GDM.

#### 3.4.3. Current consensus and mechanistic considerations

Taken together, the utility of probiotics seems limited with regard to reducing the risk of GDM, and the current best practice of diet control and exercise offers more reliable, population-wide treatment outcomes. It is important to note that some reviews and meta-analyses suggest positive effects of probiotics in GDM,^55–57^ but referenced studies have large variability in methods, metrics, and often have confounders (e.g. co-supplement of myo-inositol).^44^ Given the strong effect size of lifestyle changes in multiple studies and the mounting evidence of the dearth of probiotics’ effects in this disease, coupled with large interstudy variability, a better understanding of how gut microbiota is involved in mediating dietary management in GDM outcomes might increase the chances of targeted probiotic interventions to modulate microbiota composition and thereby potentially lead to more pronounced outcomes in the future.^58^

#### 3.4.4. Sample size, duration, and limitations

It must be highlighted that while some studies had robust sample sizes and extended intervention durations, such as 271/271 placebo/control participants over 14 weeks^42^ and 110 participants over 26 weeks,^45^ no significant differences in GDM incidence were observed. These findings emphasize that even well-powered studies with longer exposures may yield limited clinical effects.

### 3.5. Mental health

#### 3.5.1. Overview of available evidence

Limited clinical data is available on the use of probiotics during pregnancy and the impact on women’s mental health. In the few published clinical trials, there have been inconsistent outcomes potentially resulting from the use of different probiotic strains, limited samples sizes, methodological differences, and differences in the timing and length of probiotic intervention (Table 1).^47,59,60^

#### 3.5.2. Pilot and small-scale studies

Browne et al. (2021)^46^ executed a pilot RCT with 40 pregnant women and reported no significant improvement in maternal mood or psychological distress in the probiotic group supplemented with *B. animalis* subsp. *lactis* (BB-12®), and *L*. *rhamnosus* (GG®), compared to the control. This study was limited by the study design, particularly the sample size.

#### 3.5.3. Moderate- to large-scale trials

Hulkkonen et al.^48^ demonstrated a modest impact on depressive symptoms in a RCT with 430 overweight pregnant women with an intervention of *L*. *rhamnosus* (HN001), *B. animalis* subsp. *lactis* 420, and fish oil; however, the most successful clinical study was reported by Slykerman et al.^49^ Indeed, in a double-blind, multi-center RCT including 433 women, supplementations with *L*. *rhamnosus* (HN001) during and after pregnancy resulted in significantly lower prevalence of symptoms of depression and anxiety postpartum. A strength of this study was the sample size, and researchers suggested potential mechanisms of action are associated with gamma-aminobutyric acid (GABA) receptor expression, thereby alleviating anxiety-associated behavior (Figure 2).

#### 3.5.4. Meta-analyses and conflicting outcomes

In line with this, a recent systematic review and meta-analysis^60^ including 16 trials (>946 pregnant women) showed that probiotic supplementation reduced anxiety in pregnancy (*p* = 0.04) but had no effects on depression (*p* = 0.20). Nonetheless, another systematic review,^59^ including three RCTs with low risk of bias (over 713 participants), as reported using the GRADE (Grading of Recommendations Assessment, Development and Evaluation) framework, reported no impact of probiotics on “global” mental health or depression scores. However, an association between probiotic supplementation and reduced anxiety symptoms was observed. More specifically, Dawe et al. (2020) randomized 230 pregnant women with obesity (BMI ≥ 30.0 kg/m2) into study two arms: 1) placebo and 2) probiotic combination of *B. animalis* subsp. *lactis* (BB-12®) and *L*. *rhamnosus* (GG®). Depression, anxiety, and functional health and well-being were assessed at baseline in the first (12 − 17 weeks) and third (36 weeks of pregnancy) trimesters. Results showed that probiotics did not modulate or improve mental health outcomes.^47^

#### 3.5.5. Summary of study design limitations

It must be highlighted that mental health studies varied considerably in sample size, dosage, supplemented strain, and duration, affecting comparability. While some trials involved small cohorts and shorter interventions, such as 20 participants over 10 weeks,^46^ others were more robust, including up to 423 participants over 15 weeks^49^ or even 10 months.^48^ Despite these strengths, results were inconsistent, with some studies showing improvements in depression or anxiety, while others reported no significant effects.

### 3.6. Preterm birth

#### 3.6.1. Overview of clinical trials

Multiple clinical studies have been performed with probiotic intervention to modulate vaginal microbiota and related pregnancy outcomes. The vaginally applied probiotic *L*. *crispatus* (CTV-05) was shown to be safe and well accepted and tolerated in pregnant women at high risk for PTB.^50^ An overall systematic review and meta-analysis assessing pregnancy outcomes in women taking pro- and prebiotics,^61^ including five studies on probiotics in the context of very PTB (<34 weeks of gestation) and 11 studies on probiotics and PTB (<37 weeks of gestation), concluded that there was no evidence for effects on the risk of PTB or other adverse pregnancy outcomes.

#### 3.6.2. Meta-analysis limitations and interpretation

One limitation of that analysis^61^ though was that only one of the included studies truly targeted the prevention of PTB, whereas most others had different primary endpoints and only reported PTB as a secondary measure. The timing and duration of the probiotic interventions also varied among studies, making direct comparison difficult. Although some promising data suggest potential beneficial effects of probiotic supplementation during pregnancy in reducing the risk of PTB, further studies are needed to confirm these findings.

#### 3.6.3. Clinical findings: oral vs. vaginal probiotic administration

Oral supplementation of *L*. *rhamnosus* (GR-1®) and *L*. *reuteri* (RC-14®) over 12 weeks in pregnant women resulted in slight differences in interleukin-4 and interleukin-10 production compared to placebo supplementation at 28 weeks of gestation.^52^ However, these differences were not significant at 35 weeks, and there were no significant changes in vaginal microbiota either.^32^ Therefore, oral supplementation might have more indirect effects compared to vaginal probiotic administration. Importantly, pregnant women with vaginal microbiota shifts and low abundance of lactobacilli might particularly benefit from a probiotic intervention. A small study showed that gestational age and neonatal birth weight were significantly higher in a subgroup of women lacking lactobacilli in the vaginal microbiota who received vaginal *L*. *rhamnosus* (Lcr regenerans) supplementation for 8 d, although there was no difference in the PTB rate.^51^

#### 3.6.4. Antibiotic prophylaxis

Finally, vaginal probiotics might be particularly relevant in the context of antibiotic prophylaxis. A recent systematic review and meta-analysis concluded that gestational age at delivery and infant birth weight were increased, and the rate of neonatal intensive care unit (NICU) admission was reduced after vaginal probiotics administration in combination with prophylactic antibiotics versus antibiotics alone in women with preterm premature rupture of membranes.^44,62^

#### 3.6.5. Study design and comparison limitations

It is important to acknowledge that studies assessing PTB varied significantly in probiotic dosage, sample size, target site (oral, vaginal) and intervention duration, limiting the comparability of their findings. While Bayar et al. included a large cohort of over 2,000 participants,^50^ other trials involved fewer than 100 participants.^51,^ Similarly, treatment durations ranged from just 8 d^51^ to 12 weeks. Despite these differences, most studies did not report significant effects on preterm birth, underscoring the need for more consistent methodologies and longer-term trials.

### 3.7. Probiotic use during pregnancy: maternal and infant safety risks

Although microbial shifts have been implicated in various pregnancy complications, and specific bacterial taxa have even been identified as potential contributors,^5^ clinical trials testing individual probiotic strains, combinations of strains, or especially “next-generation” probiotics in pregnant women remain extremely limited.^5^

To date, most RCTs with maternal probiotic supplementation have not been associated with adverse effects to the mother or fetus,^63,64^ and there is no evidence that probiotics cross the placental barrier. However, bacterial metabolites and cell components do reach the fetus, prompting clinicians to adopt a cautious approach to supplementation. This caution is further underscored by the potential for rare but serious side effects including sepsis, bacteremia, and allergy (typically to the vehicle)^65^ along with mild but bothersome gastrointestinal symptoms including bloating, gas, and diarrhea. These risks must be weighed against the substantial evidence indicating that safety is strain-specific, only certain probiotic strains, rather than probiotics as a whole, are generally recognized as safe (“GRAS”), along with the associated benefits reviewed herein.

While concerns persist about long-term developmental effects or disturbance to natural microbiota colonization, the few meta-analyses exploring safety in neonates or infants do not support these concerns.^64,66^ Similarly, the risks of probiotic supplementation during pregnancy are akin to a number of other interventions or behaviors that affect the microbiota of the mother and/or neonate, including diet, birth mode, birth location, drug and antibiotic use, and infant feeding mode. Longer-term follow-ups are crucial to better understand the implications of maternal probiotic use and to confirm their GRAS status, ultimately addressing any remaining concerns for clinicians and pregnant women alike.^65,67,68^

Therefore, while current evidence supports the safety of specific probiotic strains during pregnancy, with many classified as GRAS and not linked to adverse maternal or neonatal outcomes, caution remains warranted due to rare but serious risks such as sepsis or allergic reactions. Although one study reported no adverse effects in children followed up to 6 y after maternal probiotic use during gestation,^69^ long-term developmental outcomes remain underexplored. This highlights the need for well-designed longitudinal studies and ongoing post-marketing surveillance to ensure safety across populations. Overall, the evidence supports a cautiously optimistic view of probiotic use in pregnancy, emphasizing the importance of strain-specific selection and continued monitoring.

## 4. Perspective on the use of probiotics before and/or during pregnancy to support maternal-fetal health and prevent pregnancy complications

The maternal microbiota in the gut, the oral cavity, and the vagina undergo significant changes during pregnancy. A substantial body of scientific literature supports the maternal microbiota as a target for intervention during pregnancy to prevent pregnancy complications. Importantly, for effective probiotic interventions, the specific microbial community at the target site of action needs to be taken into account.^70^ For example, evidence suggests that the maternal gut microbiota can support a healthy pregnancy when it has a high alpha diversity and is enriched in SCFA-producing bacteria such as bifidobacteria.^71^ A healthy vaginal microbiota during pregnancy typically exhibits low microbial diversity with predominance of *Lactobacillus* species capable of producing lactic acid and other antimicrobial compounds.^72^ Finally, beneficial features of a healthy oral microbiome during pregnancy are resistant to microbial shifts and the proliferation of opportunistic pathogens such as *F. nucleatum* and *P. gingivalis*.^73^ Thus, probiotic strains with particular benefits during pregnancy should be selected based on their specific characteristics and potential to work through a defined mode of action at the target site to ultimately support a healthy pregnancy.

As reviewed here, in a few specific cases, probiotic intervention during pregnancy has been clinically proven to support maternal health and prevent pregnancy complications. However, to date, overall clinical evidence is limited and variable. This variability is due to many challenges with probiotic intervention studies such as limited knowledge on optimal probiotic strain selection criteria, intervention periods, doses, and host factor interactions (e.g. genetic) that can influence clinical outcomes.

Further complicating research on probiotics efficacy are the cohort-specific microbiota dynamics and features that can vary more geographically than between host phenotypes. Future clinical studies will benefit from clearly defined probiotic properties, or even a shift to postbiotics, to target relevant mechanisms. Strict control of variables, along with stratification of target populations, will help clarify mechanisms of action. This approach can also account for non-responders and support more meaningful clinical outcomes.

To our knowledge, the clinical efficacy of probiotics in alleviating constipation during pregnancy is under-researched, with only two relevant studies identified.^30,31^ Additionally, many interventions with probiotics supplementation during pregnancy have relatively short periods of exposure and follow-up, making it difficult to assess long-term benefits (and risks) in participants.^38^ Thus, there is a need for larger, well-designed, well-controlled RCTs to accurately assess the effectiveness of specific probiotic interventions during pregnancy, including in some areas that have not been studied extensively so far, such as preeclampsia and other hypertensive disorders. Additionally, probiotics as a preventative measure rather than a reactionary one should also be considered in high-risk patients.

## 5. Conclusion

This review underscores the significant potential of targeting maternal microbiota with probiotics during pregnancy. While the empirical evidence shows mostly non-significant effects, the interest in this area remains high due to the critical role the microbiota plays in maternal and fetal health. The variability in study outcomes can largely be attributed to technical limitations such as small sample sizes, short follow-up periods, heterogenous target populations, and a lack of precision in probiotic strain selection or dosage tailored to specific mechanisms and sites of action. To move this field forward, future clinical studies should be conducted with optimized planning, interventions, and reporting such as:

1. Investigating the underlying biological mechanisms with a multidisciplinary approach to better understand how specific microbiota dynamics and probiotics influence pregnancy outcomes.
2. Clearly defining clinical outcome(s) and biological marker(s) informed by pre-clinical evidence and large-cohort meta-analyses.
3. Designing larger, long-term intervention clinical trials that are adequately powered to detect clinically meaningful effects.
4. Developing standardized protocols for probiotic interventions, such as dose and strain definition, to enable more consistent and comparable results across studies.
5. Systematic reporting of protocol, adverse events, and positive and negative clinical trial outcomes.
6. Exploring the use of not only single strain but multi-strain probiotics or synbiotics to enhance therapeutic outcomes, given their potential for synergistic effects.
7. Studying efficacy in diverse populations to understand if effects or absence thereof are generalizable.

Importantly, it is crucial to proceed with caution, ensuring rigorous evaluation of the safety and efficacy of probiotic products before widespread usage in pregnant populations is endorsed. Ultimately, by addressing these challenges and building on the existing knowledge base, the field can advance toward more targeted and effective probiotic interventions that are both safe and beneficial for pregnant women and their babies.

## Abbreviations

ISAPP: The International Scientific Association for Probiotics and Prebiotics
PTB: Pre-term birth
GDM: Gestational diabetes mellitus
PE: Pre-eclampsia
GBS: Group B *Streptococcus*
RCT: Randomised ontrolled rial
PPAR-γ: Peroxisome proliferator ctivated receptor-gamma
CD14: Cluster of differentiation 14
SCFAs: Short hain fatty acids
CFU: Colony forming units
GABA: Gamma-aminobutyric acid
